# A Rewired NADPH-Dependent Redox Shuttle for Testing Peroxisomal Compartmentalization of Synthetic Metabolic Pathways in *Komagataella phaffii*

**DOI:** 10.3390/microorganisms13010046

**Published:** 2024-12-30

**Authors:** Albert Fina, Sílvia Àvila-Cabré, Enrique Vázquez-Pereira, Joan Albiol, Pau Ferrer

**Affiliations:** Department of Chemical, Biological and Environmental Engineering, Universitat Autònoma de Barcelona, Carrer de les Sitges, s/n, 08193 Bellaterra, Catalonia, Spain; albertfina@gmail.com (A.F.); silvia.avila@uab.cat (S.À.-C.); enrique.vazquez@uab.cat (E.V.-P.); joan.albiol@uab.cat (J.A.)

**Keywords:** peroxisome, metabolic engineering, 3-hydroxypropionic acid, *Pichia pastoris*, *Komagataella phaffii*, acetyl-CoA, NADPH, redox shuttle

## Abstract

The introduction of heterologous pathways into microbial cell compartments offers several potential advantages, including increasing enzyme concentrations and reducing competition with native pathways, making this approach attractive for producing complex metabolites like fatty acids and fatty alcohols. However, measuring subcellular concentrations of these metabolites remains technically challenging. Here, we explored 3-hydroxypropionic acid (3-HP), readily quantifiable and sharing the same precursors—acetyl-CoA, NADPH, and ATP—with the above-mentioned products, as a reporter metabolite for peroxisomal engineering in the yeast *Komagataella phaffii*. To this end, the malonyl-CoA reductase pathway for 3-HP production was targeted into the peroxisome of *K. phaffii* using the PTS1-tagging system, and further tested with different carbon sources. Thereafter, we used compartmentalized 3-HP production as a reporter system to showcase the impact of different strategies aimed at enhancing the peroxisomal NADPH pool. Co-overexpression of genes encoding a NADPH-dependent redox shuttle from *Saccharomyces cerevisiae* (*IDP2*/*IDP3*) significantly increased 3-HP yields across all substrates, whereas peroxisomal targeting of the *S. cerevisiae* NADH kinase Pos5 failed to improve 3-HP production. This study highlights the potential of using peroxisomal 3-HP production as a biosensor for evaluating peroxisomal acetyl-CoA and NAPDH availability by simply quantifying 3-HP, demonstrating its potential for peroxisome-based metabolic engineering in yeast.

## 1. Introduction

The yeast *Komagataella phaffii* (syn. *Pichia pastoris*) has been widely used as a model organism to study peroxisomes at the genetic and molecular levels [1]. Peroxisomes, whose proliferation is induced during growth on methanol or oleic acid, contain essential enzymes involved in the metabolic pathways for the utilization of these carbon sources. For this reason, *K. phaffii* strains with deficient peroxisomal assembly are unable to grow on such substrates. This phenotypic trait was used to identify genes responsible for the correct assembly of peroxisomes and the ones enabling the transport of proteins into the peroxisomal lumen [2]. Moreover, human genes homologous to the *K. phaffii* genes responsible for peroxisomal biogenesis were identified and used to extrapolate the information on peroxisomal biogenesis mechanisms from lower to higher eukaryotes.

Peroxisomes are subcellular organelles involved in a variety of metabolic functions. In methylotrophic yeasts, such as *K. phaffii*, these organelles harbour essential enzymes for the initial oxidation of methanol and detoxification processes, notably alcohol oxidases and catalases [3]. Additionally, β-oxidation of fatty acids is confined to these compartments, allowing yeasts to metabolize both saturated and unsaturated fatty acids, producing intraperoxisomal acetyl-CoA and NADH [4]. Acetyl-CoA, a central metabolite in numerous metabolic pathways, is exported from the peroxisomal lumen to the mitochondrial matrix via two transport systems. In the glyoxylate cycle, which occurs partially in the peroxisomes, acetyl-CoA is converted into C_4_ dicarboxylic acids that subsequently enter the mitochondria. Alternatively, acetyl-CoA is converted into acetylcarnitine by carnitine acetyl-transferase (Cat2), allowing its transport across the peroxisomal membrane. Once in the cytosol or mitochondria, the reverse reaction is catalyzed by cytosolic Yat2 or Yat1, or by mitochondrial Cat2, releasing acetyl-CoA [5,6,7].

In recent years, the implementation of heterologous pathways in yeast organelles has attracted increasing attention [8,9,10,11,12]. For instance, since β-oxidation of fatty acids occurs in peroxisomes, these organelles have been harnessed to produce acyl-CoA-derived chemicals, such as medium-chain fatty acids (MCFAs), fatty alcohols, alkanes, and olefins [8,13].

While most studies on the metabolic engineering of peroxisomes focus on *Saccharomyces cerevisiae*, a few have explored the potential of *K. phaffii* peroxisomes for producing value-added chemicals. Early research reported the production of polyhydroxyalkanoates (PHA) in *K. phaffii* using glucose and oleic acid as co-substrates, achieving a final intracellular PHA weight of 1.0% [14]. Carotenoid pathway enzymes have also been successfully targeted to *K. phaffii* peroxisomes and shown their functionality using different carbon sources [15,16,17]. Additionally, simultaneous production of α-farnesene in both the cytosol and peroxisomes of *K. phaffii* led to 2.1-fold higher titers than those achieved by strains producing α-farnesene solely in the cytoplasm [18]. Recently, a multi-step pathway resembling the Calvin–Benson–Bassham cycle has been engineered into *K. phaffii* peroxisomes by targeting the enzymes with PTS1 signals, enabling this yeast to grow autotrophically on CO_2_ [19]. Furthermore, this strain was used as a platform chassis to produce organic acids (i.e., lactic acid and itaconic acid) [20]. Other products such as nucleoside analogues and sesquiterpenes have also been produced lately in peroxisome-engineered *K. phaffii* [21,22].

Peroxisomes offer significant potential advantages in metabolic engineering for several reasons: (i) They can proliferate and occupy up to 80% of the cell volume under certain conditions, such as growth on methanol or fatty acids [23], providing a large compartment for introducing synthetic pathways [24]. (ii) Protein targeting is straightforward using PTS1 or PTS2 sequences [25,26]. (iii) Co-localizing enzymes within smaller compartments increases enzyme concentrations, leading to higher reaction rates [8]. (iv) Physically separating enzymes into a different compartment prevents competition between the recombinant and endogenous pathways for the same substrates [27]. (v) It protects essential enzymes in other compartments from interacting with toxic intermediates [27]. (vi) Acetyl-CoA and NADH, key precursors for numerous metabolic engineering applications, are readily produced in peroxisomes by the abovementioned pathways. (vii) The permeability properties of the single peroxisomal membrane allow free movement of small-molecule solutes through nonselective channels while isolating metabolic intermediates or products from the cytosol [28].

The peroxisomal membrane does not allow the free diffusion of ‘bulky’ compounds such as ATP and cofactors like NAD(H), NADP(H), and CoA under in vivo conditions. However, these organelles possess specific transporter(s) for cytoplasmic ATP import (Ant1) [29] as well as CoA derivative export (i.e., the carnitine system and the glyoxylate shunt) [5,6]. Redox cofactors NAD(H) and NADP(H) are not physically exchanged between the cytoplasm and the peroxisome lumen. As a result, peroxisomes have their own pool of cofactors and redox shuttle systems allowing the transmembrane transfer of electron carriers [6,30]. In *S. cerevisiae*, two NADH-dependent redox shuttles have been identified as crucial for maintaining the intraperoxisomal redox balance through the reoxidation of NADH under various growth conditions. These are the malate/oxaloacetate (Mdh3) [6,31] and the glycerol-3-phosphate dehydrogenase 1 (Gpd1) [32] shuttles. On the other hand, *S. cerevisiae* contains a peroxisomal NADP^+^-dependent isocitrate dehydrogenase (Idp3) that catalyzes the oxidation of isocitrate to α-ketoglutarate, yielding NADPH. After that, α-ketoglutarate is exported to the cytoplasm where it is re-reduced to isocitrate by the cytosolic isocitrate dehydrogenase (Idp2), establishing a putative NADPH-regenerating redox system, which is ATP-neutral. The exchange of redox equivalents between the two compartments takes advantage of the permeability of the peroxisomal membrane to small molecules, as both α-ketoglutarate and isocitrate can freely cross the peroxisomal membrane [33,34]. Thereby, peroxisomal targeting of metabolic pathways requiring acetyl-CoA and NAD(P)H as precursors poses a significant challenge. Studies exploring cofactor availability in the peroxisome to further improve the production of value-added chemicals are still scarce [35,36].

In this study, we introduced the malonyl-CoA reductase pathway for the production of the platform chemical 3-hydroxypropionic acid (3-HP) [37], which requires acetyl-CoA and NADPH, in the peroxisome of *K. phaffii* to serve as a test bench for metabolic engineering strategies aimed at boosting peroxisomal production of compounds derived from the same key precursors. Yeast peroxisomal membranes are permeable to small metabolites [38], which can cross the membrane through pore-forming proteins, with transport occurring in a size-dependent manner [28,39]. This property likely enables the free movement of 3-HP, facilitating its subsequent transport to the extracellular space. Consequently, product quantification is simplified compared to the more complex methods required for measuring fatty acids and fatty alcohols [40,41]. Furthermore, 3-HP cannot be used as a carbon source by *K. phaffii* [42]. Therefore, 3-HP makes it an attractive reporter metabolite candidate for investigating peroxisomes as compartmentalized cell factories.

## 2. Materials and Methods

### 2.1. Construction of Strains

The plasmids and the strains used for this study are listed in Table 1. A detailed description of the molecular biology protocols to obtain each plasmid can be found in Supplementary File S1.

*K. phaffii* (*P. pastoris*) X-33 (Invitrogen-Thermo Fisher Scientific, Waltham, MA, USA) was used as a parental strain. Electrocompetent cells were obtained using a previously described protocol [43]. Plasmid DNA was linearized as follows: pBIZi_pGAP_MCR_NC_PTS1 was cut with AvrII; BB3eH_ACC_PTS1 was cut with PmeI; and BB3rN_POS5_PTS, BB3rN_cPOS5, and BB3aK_IDP2/IDP3 were cut with AscI. The restricted DNA was run on an agarose gel and the appropriate band was purified. An amount of 200 ng of linear DNA were transformed into an 80 µL aliquot of electrocompetent *K. phaffii* cells. Electroporation was performed using a Gene Pulser MXCell Electroporation System (Biorad, Hercules, CA, USA). Right after the pulse, the cells were incubated for 2 h at 30 °C with vigorous shaking. Finally, the cells were plated on YPDagar medium (1% yeast extract, 2% peptone, 2% glucose, 1.5% agar) supplemented with the appropriate antibiotic (100 µg/mL Zeocin, 200 µg/mL Hygromycin, 500 µg/mL Geneticyn, or 100 µg/mL clonNAT). All antibiotics were purchased from Invivogen (San Diego, CA, USA). The plates were incubated for 48 h at 30 °C.

Twelve clones were re-streaked on YPD supplemented with the appropriate antibiotic and incubated for 48 h and 30 °C. Positive clones were checked by colony PCR.

**Table 1:** Plasmids and *K. phaffii* strains used in this study.

Plasmid Name	Expression Cassettes in Each Plasmid	Source
pBIZi_pGAP_MCR_NC	P*_GAP_*-*mcr*_Ca_(N-ter)-AOX1tt P*_GAP_*-*mcr*_Ca_(C-ter)-AOX1tt	[42]
pBIZi_pGAP_MCR_NC_PTS1	P*_GAP_*-*mcr*_Ca_(N-ter)_PTS1-AOX1tt P*_GAP_*-*mcr*_Ca_(C-ter)_PTS1-AOX1tt	This study
BB3eH_ACC1	P*_GAP_*_-_*ACC1_Y_*_l_(Δ*Bbs*I)-ScCYC1tt	[42]
BB3eH_ACC1_PTS1	P*_GAP_*_-_*ACC1_Y_*_l_(Δ*Bbs*I)_PTS1-ScCYC1tt	This study
BB1_12_pGAP		[44]
BB1_23		[44]
BB1_34_RPS3tt		[44]
BB1_23_cPOS5	*cPOS5_Sc_*	[42]
BB3rN_14		[44]
BB3rN_POS5_PTS	P*_GAP_*_-_*POS5_Sc__PTS1*-RPS3tt	This study
BB3rN_cPOS5	P*_GAP_*_-_*POS5_Sc_*-RPS3tt	This study
BB3aK_AC		[44]
BB3aK_IDP2/IDP3	P*_TEF2_*_-_ *IDP2_Sc_*-RPS3tt P*_MDH3_*-*IDP3*_Sc_-ScCYC1tt	This study
**Strain name**	**Parental strain +** **New plasmid integration**	**Source**
X-33		Invitrogen—Thermo Fisher Scientific
PpM	X33 + pBIZi_pGAP_MCR_NC_PTS1	This study
PpMA	PpM + BB3eH_*ACC1*_PTS1	This study
PpMAP	PpMA+ BB3rN_*POS5*_PTS	This study
PpMAS	PpMA + BB3aK_*IDP2*/*IDP3*	This study
PpMAC	PpMA + BB3rN_c*POS5*	This study
PpMASC	PpMAS + BB3rN_c*POS5*	This study
PpHP2	X33 + pBIZi_pGAP_MCR_NC	[42]
PpHP4	PpHP2 + BB3eH_*ACC1*	[42]
PpHP6	PpHP2 + BB3eH_*ACC1*_c*POS5*	[42]

### 2.2. Screening Conditions

Buffered Minimal medium (100 mM potassium phosphate buffer pH 6, 1.34% yeast nitrogen base with ammonium sulphate without amino acids (Becton Dickinson Difco™, Franklin Lakes, NJ, USA), 0.4 mg/L biotin) was supplemented with the appropriate carbon source: glucose (BMD; 20 g/L of glucose), glycerol (BMG; 1% *v*/*v* glycerol), methanol (BMM; 0.5% *v*/*v* methanol), or oleic acid (BMO; 1% *v*/*v* oleic acid and 0.5% *v*/*v* Tween80).

Cells were cultured overnight on 5 mL of YPD (1% yeast extract, 2% peptone, 2% glucose) in 50 mL falcon tubes at 30 °C and 200 rpm. The day after, 24-deep-well plates containing 2 mL of medium (BMD, BMG, BMM, or BMO) were inoculated at a starting OD_600_ of 0.1 and incubated at 25 °C and 220 rpm in a Multitron Standard incubator shaker (Infors HT, Bottmingen, Switzerland) with a 2.5 cm orbit using a platform with a slope of 20°. The cultures on BMD or BMG were incubated for 48 h. The cultures on BMO were incubated for 72 h. In the case of BMM, a pulse of 1% *v*/*v* methanol was performed on each culture after 24 h of cultivation. The BMM cultures were harvested after 48 h of incubation.

### 2.3. Analytical Methods

At the end of the cultures, 2 mL samples were centrifuged at 12,000× *g* for 10 min. The supernatants were filtered through a 0.22 µm filter (Millex SLLGX13, Millipore, CA, USA).

Full consumption of the substrates was checked using a previously described HPLC protocol [42]. An HPLC Ultimate3000 (Dionex—Thermo Fisher Scientific) with a UV detector at 210 nm and a Refractive Index (RI) detector (Dionex—Thermo Fisher Scientific) was used together with an ionic exchange column ICSep ICE-COREGEL 87H3 (Transgenomic, Omaha, NE, USA) to separate the compounds of the supernatant. The flow rate of the mobile phase (6 mM sulphuric acid) was set to 0.6 mL/min and the injection volume was set to 20 µL. The 3-HP produced by the cytosolic variants (PpHP strains) was quantified from the RI spectrum.

For 3-HP quantification in samples derived from the peroxisomal variants constructed in this study, a previously described HPLC-MS method was used [42]. A Prominence HPLC (Shimadzu, Kyoto, Japan) with a single-quadrupole Shimadzu-2010A mass spectrometry (MS) detector with an electrospray ionization source was used. An ICSep 87H USP L17 column (Transgenomic, NE, USA) was used to separate the metabolites of the supernatant. The flow rate of the mobile phase (16 mM formic acid) was set to 0.15 mL/min. The injection volume was set to 2 µL. The MS analyser was set to 89 *m*/*z* for negatively charged molecules. The detector settings were as follows: curved desolvation line (CDL) temperature at 200 °C, heat block temperature at 200 °C, voltage of the detector at 1.5 kV, nebulizing gas (nitrogen) flow at 1.5 L/min, and drying gas (nitrogen) flow at 10 L/min. All samples were analyzed in duplicate.

## 3. Results and Discussion

### 3.1. Construction of K. phaffii Strains Engineered for 3-HP Production in Peroxisomes

**Figure 1:**
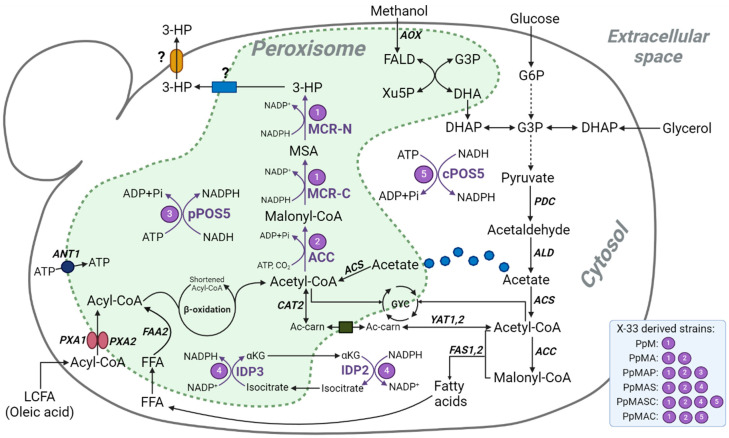
Metabolic pathway engineering for peroxisomal 3-HP synthesis in *K. phaffii* from various carbon sources. Heterologous and endogenous genes are presented in purple and black, respectively. MCR-C and MCR-N, C- and N-terminal domains of malonyl-CoA reductase, respectively; *ACC*, acetyl-CoA carboxylase; *POS5*, NADH kinase; *IDP2* and *IDP3*, NADP-dependent isocitrate dehydrogenases isoenzymes; *AOX*, alcohol oxidase; *PDC*, pyruvate decarboxylase; *ALD*, acetaldehyde dehydrogenase; *ACS*, acetyl-CoA synthetase; *YAT1,2* and *CAT2*, carnitine acetyl-transferases; *FAS1,2*, fatty acid synthases; *FAA2*, acyl-CoA synthetase; *ANT1*, ATP transporter; *PXA1,2*, ABC transporter family D; FALD, formaldehyde; Xu5P, xylulose-5-phosphate; G3P, glyceraldehyde-3-phosphate; DHA, dihydroxyacetone; DHAP, dihydroxyacetone phosphate; G6P, glucose-6-phosphate; GYC, glyoxylate cycle; Ac-carn, acetylcarnitine; MSA, malonate semialdehyde; 3-HP, 3-hydroxypropionic acid; αKG, α-ketoglutarate; FFA, free fatty acids; LCFA, long-chain fatty acids.

In a previous study, we showed that the individual expression of the two enzymatic domains of the bi-functional malonyl-CoA reductase from *Chloroflexus aurantiacus* (MCR C- and N-terminal), catalyzing two consecutive NADPH-consuming reactions converting malonyl-CoA into 3-HP, leads to higher 3-HP production in the cytosol of *K. phaffii* compared to the expression of the undissected *mcr*_Ca_ gene [42]. Following up on this, the genes encoding for the MCR C- and N-terminal domains were expressed under the control of the *GAP* promoter (p*GAP*) and tagged with the PTS1 C-terminal sequence for its compartmentalization in peroxisomes, resulting in strain PpM (Figure 1). Aiming at increasing the availability of the pathway precursor malonyl-CoA, the acetyl-CoA carboxylase (*ACC1*) gene from *Yarrowia lypolitica* fused to a PTS signal encoding sequence was expressed using the p*GAP* in strain PpM, obtaining strain PpMA.

The second precursor limiting 3-HP production via the malonyl-CoA pathway is NADPH. In the present work, we have studied different strategies aiming at increasing this cofactor’s delivery to the *K. phaffii* peroxisome. Two independent mechanisms were considered: First, a mitochondrial NADH kinase from *S. cerevisiae* (*POS5*) was expressed under the control of p*GAP* and targeted to the peroxisome of strain PpMA by substituting the mitochondrial tag sequence with PTS1 (p*POS5*), resulting in strain PpMAP. Pos5 catalyzes the ATP-dependent conversion of NADH into NADPH. Ectopic expression of the cytosolic-targeted version of *POS5* (c*POS5*) in *K. phaffii* has been proven to increase the NADPH/NADP^+^ ratio, thereby increasing the production of heterologous proteins and bulk chemicals [42,45]. A strain harbouring the c*POS5* gene was also constructed (PpMAC). Second, we considered a cyclic pathway that delivers NADPH redox equivalents from the cytosol into the peroxisome, namely the Idp2/Idp3 shuttle [33]. To date, an *IDP3* homologous gene has not been identified in the *K. phaffii* genome [46]. Thus, *IDP2* and *IDP3* genes originating from *S. cerevisiae* were heterologously expressed in PpMA using the *TEF2* and *MDH3* constitutive promoters, respectively, resulting in strain PpMAS. The original Idp3 from *S. cerevisiae* contains a PTS1 sequence tag at its C-terminal end and, therefore, is prone to being imported from the cytosol into the peroxisomal matrix of *K. phaffii*. Furthermore, the expression of the NADPH-linked redox shuttle was combined with the expression of c*POS5* in strain PpMASC, aiming at increasing the cytosolic NADPH pool and the subsequent transport flux of NADPH to the peroxisome (Figure 1).

### 3.2. Using 3-HP Produced from Oleic Acid as a Reporter Metabolite to Detect Peroxisomal NADPH Levels

Peroxisomal production of 3-HP was first investigated using an unsaturated fatty acid (oleic acid) as a substrate to ensure sufficient delivery of acetyl-CoA. Moreover, oleic acid is known to promote the proliferation of the peroxisomes. In this way, we also ensured that this organelle would have sufficient capacity to allocate the heterologous enzymes needed for 3-HP synthesis.

**Figure 2:**
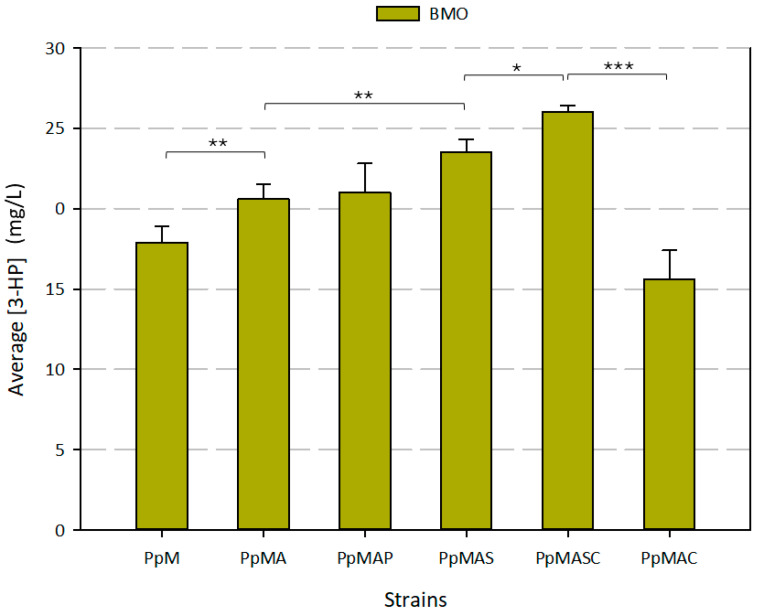
Average final 3-HP concentration produced from oleic acid by each *K. phaffii* recombinant strain. Data are presented as the mean ± SD of biological triplicates. Statistically significant differences between two groups are shown in the graph. Statistical analysis was conducted using a two-tailed unpaired Student’s *t*-test (* *p* < 0.01, ** *p* < 0.001, *** *p* < 0.0001). Raw data available in Supplementary File S2.

Three independent clones of strain PpM, engineered to express the genes encoding for the MCR C- and N-terminal domains in the peroxisome of *K. phaffii*, were grown in triplicate on Buffered Minimal medium supplemented with oleic acid (BMO) for 72 h. While peroxisomal Acc1 activity has not been conclusively confirmed in yeast, and the presence of malonyl-CoA in this organelle remains a matter of debate, strain PpM produced 17.9 ± 1.0 mg/L of 3-HP in BMO (Figure 2). We hypothesize that this significant 3-HP production stems from cytosolic malonyl-CoA, as proteins tagged with a PTS1 signal are initially synthesized and folded in the cytosol, where they transiently reside before being transported into the peroxisomal matrix.

The expression of the *Y. lipolytica* gene encoding an acetyl-CoA carboxylase (*ACC1*) targeted to the peroxisome of strain PpMA significantly increased the production of 3-HP by 15% in comparison with PpM strain (*p* < 0.001), reaching 20.6 ± 0.9 mg/L of 3-HP (Figure 2). The delivery of acetyl-CoA is ensured when oleic acid is used as a substrate, and thus the availability of this metabolite should not be a limiting factor for producing 3-HP in this strain. Hence, PpMA was considered as a suitable platform strain to test different strategies aiming at increasing NADPH peroxisomal availability, using 3-HP production as a reporter metabolite and oleic acid as reference substrate.

Expression of the gene encoding *S. cerevisiae*’s *POS5* NADH kinase targeted to the cytosol (c*POS5*) of *K. phaffii* has already shown positive effects on the production of both recombinant proteins and 3-HP [42,45]. However, when *POS5* was targeted to the peroxisome (p*POS5*) of PpMA, the resulting strain PpMAP produced 21.0 ± 1.8 mg/L of 3-HP, which was statistically comparable to the amount produced by its parental strain (Figure 2). The Pos5 NADH kinase converts NADH and ATP into NADPH and ADP. It is well reported that low peroxisomal ATP availability is another limiting factor regarding the use of the peroxisomes for metabolic engineering applications [8]. Thus, failure of this strategy to increase the NADPH availability for 3-HP production may be ascribed to its ATP dependence concomitant with the mentioned ATP limitation. Some strategies to increase the availability of ATP in the peroxisome of yeast include the overexpression of Ant1, a peroxisomal ATP transporter [35] (Figure 1). In this study, we have considered alternative strategies to increase NADPH availability in the peroxisome that do not rely on the use of peroxisomal ATP.

The *S. cerevisiae* Idp2/Idp3 shuttle was expressed in strain PpMA. Idp2 catalyzes the conversion of cytosolic α-ketoglutarate to isocitrate consuming cytosolic NADPH, while Idp3 catalyzes the production of isocitrate from α-ketoglutarate in the peroxisome, generating peroxisomal NADPH. The *K. phaffii* strain overexpressing the Idp2/Idp3 shuttle (PpMAS) produced 23.5 ± 0.8 mg/L of 3-HP. Such 3-HP production is significantly higher (13.8%, *p* < 0.001) than the amount produced by the parental strain PpMA (Figure 2). The fold change increase with the implementation of the NADPH shuttle is consistent with previous findings in *S. cerevisiae*, where the overexpression of the Idp2/Idp3 shuttle led to an increase of 15–20% in squalene production [35]. The increase in 3-HP production provides strong evidence for the peroxisomal localization of the synthetic malonyl-CoA reductase pathway. Should this route be cytosolic, enhancing peroxisomal NADPH levels would not have improved 3-HP production, as the peroxisomal membrane is impermeable to NADPH, preventing its exchange between the cytosol and this compartment.

Since the PpMAS strain shuttles NADPH redox equivalents from the cytosol into the peroxisome, we hypothesized that increasing the cytosolic NADPH/NADP^+^ ratio would boost exchange fluxes through the Idp2/Idp3 shuttle, leading to higher NADPH availability in the peroxisome and, consequently, increased 3-HP production. To this end, we overexpressed c*POS5* in the PpMAS strain. The resulting strain (PpMASC) produced 26.0 ± 0.4 mg/L of 3-HP, a significant 10.8% improvement over PpMAS (*p* = 0.003). These findings demonstrate that co-expression of cytosolic NADH kinase (cPos5) with the Idp2/Idp3 shuttle enhanced both cytosolic and peroxisomal NAPDH availability, leading to improved 3-HP production. This is consistent with the fact that strain PpMAC, which harbours the cPos5 but lacks the Idp2/Idp3 shuttle, produced significantly less 3-HP compared to strain PpMASC (15.6 ± 1.8 mg/L) (*p* < 0.0001). Moreover, strain PpMAC showed 24.2% lower 3-HP production compared to its parental strain, PpMA (Figure 2). Introduction of the cytosolic Pos5 NADH kinase in *K. phaffii* has been reported to cause an energy drain due to increased ATP consumption [45], which may explain the reduced yield in this strain. These results also suggest that *K. phaffii* lacks any major native peroxisomal NADPH-dependent redox shuttle that is metabolically active under the tested conditions.

### 3.3. Testing the Potential of Peroxisomal 3-HP Production as a Synthetic Biosensor in K. phaffii Strains Growing on Different Carbon Sources

To further investigate the applicability of peroxisomal 3-HP production as a reporter pathway when glucose, glycerol, and methanol are used as substrates, the same series of strains were cultured on Buffered Minimal medium supplemented with glycerol (BMG), glucose (BMD), or methanol (BMM).

When growing on oleic acid, acetyl-CoA is directly produced in the peroxisome through the β-oxidation of fatty acids. Conversely, the glycolysis of carbon sources such as glucose and glycerol to acetyl-CoA via pyruvate occurs in the cytosol (Figure 1). Alternatively, the mitochondrial pyruvate dehydrogenase complex can also catalyze the conversion of pyruvate to acetyl-CoA. On the other hand, methanol is first oxidized to formaldehyde by alcohol oxidase (Aox) in the peroxisome and can then be assimilated via the xylulose-5-phosphate pathway, leading to the formation of glyceraldehyde-3-phosphate (G3P), an intermediate in glycolysis. This G3P is channelled into lower glycolysis, where it can be converted into pyruvate. Importantly, acetyl-CoA cannot freely diffuse across cellular membranes, including those of the nucleus and peroxisome. Nevertheless, acetate, which can be produced from pyruvate, can readily diffuse to different cell compartments, where it can be converted into acetyl-CoA by means of acetyl-CoA synthetases (Acs) [47].

In the cytosol, acetyl-CoA is carboxylated by Acc1 in an ATP-dependent reaction to form malonyl-CoA, both of which are key precursors for fungal de novo fatty acid biosynthesis, catalyzed by cytosolic fatty acid synthases (Fas1, Fas2). Free fatty acids (FFAs) are transported across the peroxisomal membrane and subsequently activated inside the peroxisome by the acyl-CoA synthetase Faa2, whereas fatty acyl-CoA esters are taken up through the ABCD transporters Pxa1 and Pxa2 [48]. Once inside the peroxisome, these activated fatty acids undergo β-oxidation, contributing to the pool of peroxisomal acetyl-CoA (Figure 1). Interestingly, genes encoding fatty acid synthases (*FAS1*, *FAS2*) are up-regulated in methanol-grown cells, while under glucose-limited conditions, there is a strong induction of genes involved in fatty acid utilization (e.g., *FAA2*, *PXA1*, *PXA2*) [49].

**Figure 3:**
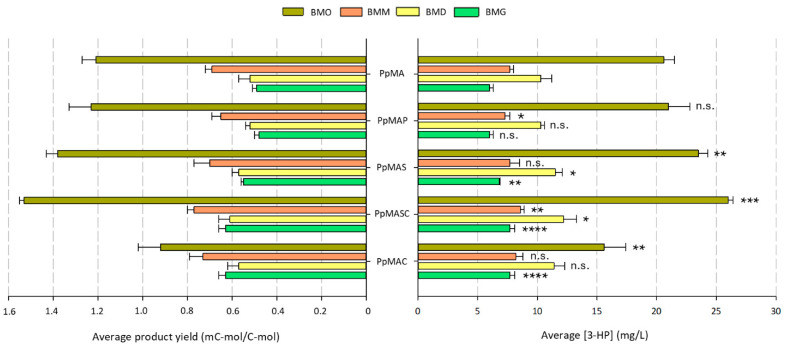
Final 3-HP concentrations and overall product yields (Y_P/S_) obtained from BMO, BMM, BMD, and BMG by platform strain PpMA and its derivative strains. Data are presented as the mean ± SD of biological triplicates. Statistically significant differences between PpMA and each derivative strain are indicated in the graph. Statistical analysis was conducted using a two-tailed unpaired Student’s *t*-test (* *p* < 0.05, ** *p* < 0.001, *** *p* < 0.0001, **** *p* < 0.00001, n.s. not significant). Raw data available in Supplementary File S2.

Several studies have highlighted the biosynthetic potential of yeasts to produce acetyl-CoA-derived compounds within peroxisomes from carbon sources such as glycerol, glucose, and methanol. These compounds include squalene [35], fatty alcohols [36,41,50], alkanes and olefins [50], terpenes [16], and polyketides [51,52]. Similarly, the malonyl-CoA-to-3-HP pathway has been confirmed to function effectively within the peroxisomal compartment. This is evidenced by the detectable production of 3-HP across all strains engineered in this study, even when using these alternative carbon sources (Figure 3).

As expected, the highest 3-HP levels were achieved in BMO, since the β-oxidation of fatty acids delivers large amounts of peroxisomal acetyl-CoA (a precursor for 3-HP) and energy. Moreover, fatty acids induce peroxisomal proliferation, increasing both the copy number and size of peroxisomes involved in the pathway. Notably, across the other culture media, the order of highest to lowest 3-HP production rank was BMD, BMM, and BMG, regardless of the strain used (Figure 3). Similar results were described by Bhataya et al. [16], with higher lycopene production in *K. phaffii* peroxisomes on glucose than on glycerol or methanol. Overall, our synthetic biosensor revealed the differences in relative peroxisomal acetyl-CoA availability under various culture conditions.

Several studies have shown how *K. phaffii*’s metabolism can adapt to the consumption of different carbon sources, highlighting the widespread effects of substrate choice at the transcriptomic level [49,53]. Thereby, the divergent behaviour of the 3-HP-producing strains growing under different carbon sources is plausible despite the expression of the same set of heterologous genes (Figure 3). Nevertheless, a few consistent trends were observed across all conditions: The final 3-HP concentrations obtained by strain PpMAP were either similar to or lower than those observed in strain PpMA, regardless of the substrate used, indicating that targeting the NADH kinase gene to the peroxisome (p*POS5*) did not enhance 3-HP production. Conversely, overexpression of the *S. cerevisiae* Idp2/Idp3 shuttle in strain PpMA, yielding strain PpMAS, significantly increased 3-HP production on all substrates except methanol, which remained unchanged (*p* < 0.001 for BMO and BMG; *p* = 0.030 for BMD). Increasing the cytosolic NADPH/NADP^+^ ratio through overexpression of c*POS5* (strain PpMAC) also failed to yield any noticeable changes on BMM and BMD, since the excess NADPH in the cytosol is unable to cross the peroxisomal membrane. Moreover, it led to a reduction in 3-HP titers on BMO compared to strain PpMA. However, combining both strategies in strain PpMASC led to a significant enhancement in 3-HP production across all substrates tested (*p* < 0.0001 for BMO; *p* < 0.001 for BMM; *p* < 0.01 for BMD; *p* < 0.00001 for BMG) (Figure 3).

Indeed, strain PpMASC consistently achieved the highest concentrations of 3-HP, with increases ranging from 10 to 30% depending on the substrate, in comparison to strain PpMA. These results confirm the beneficial effect of simultaneously harbouring the NADPH-dependent redox shuttle and the cytosolic NADH kinase. While the enhancement in 3-HP yield concomitant with the introduction of *IDP2*/*IDP3* and c*POS5* genes was confirmed regardless of the substrate used, we cannot discard the hypothesis that acetyl-CoA may become a limiting factor in strains growing on substrates such as glucose, glycerol, and methanol. Consequently, the reporting system introduced here can only act as an indicator of peroxisomal NADPH availability when cultivation media are enriched with a fatty acid source to ensure sufficient acetyl-CoA delivery through β-oxidation.

To assess the carbon utilization efficiency for 3-HP production across the different metabolic engineering strategies, we calculated the C-yield for each strain on each substrate (Figure 3). The highest yields were observed when cells were grown on oleic acid, as the carbon atoms in fatty acids are more reduced compared to those in glucose and glycerol. Strain PpMASC achieved a remarkable 3-HP yield of 1.53 ± 0.02 mC-mol/C-mol of oleic acid, the highest yield recorded among the tested strains and carbon sources.

The expression of genes encoding for the two MCR subunits, Acc1, and NADH kinase was driven by the constitutive p*GAP*. Waterham et al. [54] first identified and cloned p*GAP* to constitutively express a bacterial β-lactamase in *K. phaffii* growing on different carbon sources. Expression levels of the recombinant protein were the lowest in methanol-grown cells and the highest in glucose-grown cells, consistent with reports on reduced p*GAP* transcriptional levels under methanolic growth conditions compared to glucose or glycerol metabolism [55,56]. Interestingly, the 3-HP yields observed in this study increased by 21–34% and 16–50% in methanol versus glucose or glycerol, respectively, depending on the strain used. The average product yield from each substrate revealed statistically significant differences between BMG and BMD when compared to BMM (*p* < 0.001 and *p* < 0.0001, respectively), while yields on BMG and BMD were comparable (*p* = 0.68). A rough estimation of lycopene production yield, based on data from Bhataya et al. [16], indicated that the product yield on methanol was 1.6 to 1.7 times higher than that achieved on glucose, consistent with our findings. These results point at the beneficial role of induced peroxisome proliferation in enhancing 3-HP production within this organelle.

### 3.4. Comparison of the Peroxisomal and Cytosolic Variants of the 3-HP Producing Strains

In a previous study, we developed a series of *K. phaffii* strains expressing the cytosolic version of the malonyl-CoA pathway for 3-HP production on glycerol [42]. In strain PpHP2, individual genes encoding for each of the two domains of the malonyl-CoA reductase (MCR C- and N-terminal) were co-expressed. The cytosolic overexpression of the *ACC1* gene in strain PpHP2 resulted in strain PpHP4, whereas co-expression of both *ACC1* and c*POS5* in the parental strain PpHP2 resulted in strain PpHP6. All the expression cassettes were under the control of p*GAP*. This series of strains represents the cytosolic counterparts of strains PpM, PpMA, and PpMAC constructed in this work, respectively.

**Figure 4:**
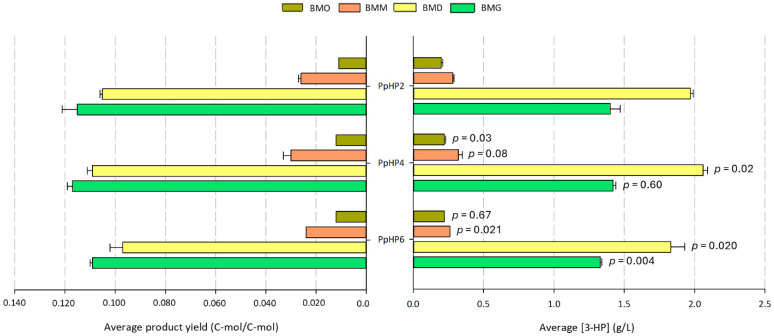
Final 3-HP concentrations and global product yields (Y_P/S_) obtained from BMO, BMM, BMD, and BMG by each cytosolic *K. phaffii* strain. Data are presented as the mean ± SD of biological triplicates. The *p*-values resulting from two-tailed unpaired Student’s *t*-tests comparing two groups (PpHP4 vs. PpHP2 and PpHP6 vs. PpHP4) on each substrate are indicated. Raw data available in Supplementary File S2.

A representative clone from each PpHP strain was cultivated in triplicate on BMG, BMD, BMM, and BMO media. Overexpression of *Y. lypolitica ACC1* in strain PpHP4 led to a statistically significant increase in 3-HP production on BMO and BMD (*p* = 0.03 and 0.02, respectively) compared to PpHP2 (Figure 4). This result aligns with the fact that substantial amounts of acetyl-CoA are generated during the β-oxidation of fatty acids, which occurs predominantly in BMO. When glucose is used as a substrate, cytosolic acetyl-CoA is produced from pyruvate, primarily via acetaldehyde and acetate [57]. The increased 3-HP production upon *ACC1* overexpression suggests that the carboxylation of acetyl-CoA to form malonyl-CoA, catalyzed by endogenous Acc1, was a rate-limiting step for 3-HP synthesis under these conditions. In fact, cytosolic Acc1 activity in yeasts is reduced through phosphorylation by Snf1 at serine residues when glucose is depleted, lowering cytosolic malonyl-CoA synthesis [58]. Conversely, glycerol-grown cultures did not show malonyl-CoA limitations for 3-HP production, as previously noted by Fina et al. [42].

Ectopic expression of c*POS5* in strain PpHP4, resulting in strain PpHP6, led to a significant reduction in 3-HP production across all substrates except for oleic acid when compared to PpHP4. Specifically, 3-HP titers decreased by 6.7%, 12.5%, and 24.6% on BMG, BMD, and BMM, respectively (*p* = 0.004, 0.020, and 0.021) (Figure 4). Pos5 overexpression increases ATP demand for consumption in the NADH kinase reaction, which drains cellular energy [45]. Additionally, the cytosolic pathway from pyruvate to acetyl-CoA requires 2 ATP equivalents, and another ATP is consumed when Acc1 converts acetyl-CoA to malonyl-CoA. We hypothesize that this cumulative energy cost negatively impacted 3-HP yield on these substrates.

Evidence for the successful translocation of the 3-HP-producing enzymes into peroxisomes can be inferred from the observation that all strains harbouring the peroxisome-targeted malonyl-CoA pathway exhibited higher 3-HP yields when grown on oleic acid and methanol compared to glycerol or glucose (Figure 3 and Figure 4), also suggesting that the proliferation of peroxisomes, which is strongly induced by oleic acid and methanol, considerably impacts 3-HP production. In contrast, the cytosolic strains (PpHP) showed the opposite trend, achieving 3-HP yields up to 4 times higher on glucose and glycerol than on methanol, and up to 10 times higher than on oleic acid. Nevertheless, the cytosolic strains still outperformed peroxisomal strains in absolute 3-HP production across all carbon sources. For instance, PpHP strains produced at least 10 times more 3-HP than peroxisomal strains in BMO, with up to a 200-fold increase in BMG. These findings provide further evidence that the PTS1-tagged enzymes recombinantly produced in the peroxisomal strains series are properly localized and do not remain in the cytosol, in contrast to the PpHP strain series, where recombinant enzymes are exclusively cytosolic.

The substantially lower 3-HP yields obtained with the peroxisome-engineered *K. phaffii* strain series compared to its cytosolic counterparts suggest potential metabolic bottlenecks remain. For instance, increasing the expression ratio of MCR-C to MCR-N to balance subunit activity has been shown to enhance 3-HP production [59,60]. In addition, the lower 3-HP titers could be attributed to transport limitations across both the peroxisomal and plasma membranes, particularly when 3-HP is predominantly in its dissociated form. This situation occurs when 3-HP is produced in the yeast peroxisomal matrix, which has a pH between 5.8 and 6.0 [61], whereas the pKa value of 3-HP is 4.51 [62]. Overexpressing monocarboxylate transporters anchored to the peroxisomal membrane might be a promising strategy to enhance 3-HP export to the cytosol. Notably, a recent study has demonstrated that overexpression genes encoding for lactate transporters in the plasma membrane of *K. phaffii* strains harbouring the synthetic b-alanine pathway for 3-HP synthesis located in the cytosol led to a 42% increase in final product titers [63].

## 4. Conclusions

In this study, we successfully localized the malonyl-CoA pathway for 3-HP production in the peroxisomes of *K. phaffii* via PTS1 protein tagging. Although 3-HP titers in peroxisome-engineered strains were considerably low, this system enabled the construction of a synthetic biosensor to assess in vivo acetyl-CoA and NADPH availability in the peroxisome. By quantifying 3-HP levels, we effectively sensed relative differences in peroxisomal acetyl-CoA availability in cells grown on different carbon sources, including peroxisome-proliferating and -non-proliferating conditions. Additionally, 3-HP served as a reporter metabolite to test two distinct strategies aimed at enhancing the peroxisomal NADPH pool in *K. phaffii*. Co-expression of the Idp2/Idp3 redox shuttle with cytosolic NADH kinase (cPos5) was shown to consistently boost 3-HP production regardless of the substrate, demonstrating the potential of the biosensor to aid peroxisome-based metabolic engineering strategies in yeast.

## Data Availability

The original contributions presented in this study are included in the article/Supplementary Material. Further inquiries can be directed to the corresponding author.

## Supplementary Materials

The following supporting information can be downloaded at https://www.mdpi.com/article/10.3390/microorganisms13010046/s1: Supplementary File S1: Detailed description for the generation of each plasmid and recombinant *K. phaffii* strain. Table S1: Primer list. Supplementary File S2: Raw data from the 24-deep-well plate cultivations.
