# Supplementary material for: A Rewired NADPH-Dependent Redox Shuttle for Testing Peroxisomal Compartmentalization of Synthetic Metabolic Pathways in Komagataella phaffii
Source: Microorganisms. 2024 Dec 30;13(1):46. doi: 10.3390/microorganisms13010046 (PMC11767246; doi:10.3390/microorganisms13010046)
Supplement: Supplementary file 1 [file microorganisms-13-00046-s001.zip › Supplementary file S1.pdf]

Supplementary Information

A rewired NADPH-dependent redox shuttle for testing peroxisomal compartmentalization of synthetic metabolic pathways in *Komagataella phaffii*

Albert Fina‡, Sílvia Àvila-Cabré‡, Enrique Vázquez-Pereira, Joan Albiol & Pau Ferrer*

‡Equal contribution

*Corresponding author

Pau.Ferrer@uab.cat

Department of Chemical, Biological and Environmental Engineering, Universitat Autònoma de Barcelona, Bellaterra, Catalonia, Spain.

Molecular biology protocols

A list of primers can be found in Table S1.

The plasmids pBIZi_pGAP_MCR_NC_PTS1 and BB3eH_ACC_PTS1 were obtained using the NEBuilder HiFi kit from New England Biolabs (NEB, MA, USA). For pBIZi_pGAP_MCR_NC_PTS1, three PCR reactions were set using the primer pairs NC_PTS_1_FW/NC_PTS_1_RV, NC_PTS_2_FW/NC_PTS_2_RV, and NC_PTS_3_FW/NC_PTS_3_RV. The plasmid pBIZi_pGAP_MCR_NC was used as a template. The three PCR products were purified from an agarose gel and ensembled using the NEBuilder HiFi kit according to the protocol provided by the manufacturer. For the BB3eH_ACC_PTS1, two PCR reactions were performed using the primer pairs ACC_PTS_1_FW/ACC_PTS_1_RV and ACC_PTS_2_FW/ACC_PTS_2_RV. The plasmid BB3eH_ACC was used as a template, and the two PCR fragments were ensembled to generate the vector BB3eH_ACC_PTS1. The ensembled DNA was transformed into electrocompetent *Escherichia coli* DH5α and plated on selective LB-medium (1% tryptone, 0.5% yeast extract, and 0.5% NaCl at pH 7.5).

To obtain the plasmids BB3rN_POS5_PTS1, the PTS1 sequence was added to the C-terminus of cPOS5 using the primer pairs POS5_PTS_FW/POS5_PTS_RV. The plasmid BB1_23_cPOS5 was used as a template. The resulting amplicon was cloned into BB1_23 using the restriction enzyme BsaI-HFv2 from NEB to generate the plasmid

BB1_23_POS5_PTS1. A previously described Golden Gate protocol was used to generate BB3rN_POS5_PTS1 and BB3rN_cPOS5. For BB3rN_cPOS5, the plasmids BB1_12_pGAP, BB1_23_cPOS5, BB1_34_RPS3tt, and BB3rN_14 (the destination plasmid) were added to the Golden Gate reaction containing BbsI-HF from NEB. To generate BB3rN_POS5_PTS1, the same plasmids was used, but the plasmid BB1_23_cPOS5 was substituted by BB1_23_POS5_PTS1. The resulting plasmids were transformed into chemocompetent *E. coli* DH5α and plated on selective LB-medium.

The DNA sequences of *IDP2* and *IDP3* from *Saccharomyces cerevisiae* were codon optimized by Integrated DNA Technologies (IA, USA). The sequence *IDP3* already contains a peroxisomal tag-sequence at the C-terminus of the gene. The sequence of the pTEF2 promoter and RPS3tt terminator were added to *IDP2*, and the sequence of the pMDH3 promoter and ScCYCtt were added to *IDP3*. BsaI restriction sites were added at each end of the linear DNA. Each DNA sequence for each expression cassette was bought as a gBlock to Integrated DNA Technologies. The two linear DNA sequences were simultaneously cloned into BB3aK_AC using the aforementioned Golden Gate protocol with BsaI-HFv2 to obtain the plasmid BB3aK_IDP2/IDP3. The resulting plasmid was transformed into chemocompetent *E. coli* DH5α and plated on selective LB-medium

For all plasmids, several clones were screened by checking their restriction pattern. The integrity of the sequence of the correct clones was checked by Sanger sequencing at the Genomics and Bioinformatics Service at the Institut de Bioquímica i Biotecnologia of the Universitat Autònoma de Barcelona (Bellaterra, Spain).

Table S1: Primers list.

Primer name	Sequence
NC_PTS_1_FW	TGCGCGGAACCCCTATTTGTTTA
NC_PTS_1_RV	GAAATCACTTTGAACATCCCAGCTAACATCTCTAAGTTGTAATAAGA GCTCGAGACCACTAGTACGGGCCCT
NC_PTS_2_FW	ACAAATAGGGGTTCCGCGCAGATCTTTTTTGTAGAAATGTCTTGG
NC_PTS_2_RV	AGGGCCCGTACTAGTGGTCTCGAGCTCTTACAACTTAGAAACAGTG ATTGCTCTACCTCTATGGATTCT

NC_PTS_3_FW	GAAATCACTTTGAACATCCCAGCTAACATCTCTAAGTTGTAATAAGA GCTCGAGACCACTAGTACGGGCCCT
NC_PTS_3_RV	TAAGAGCTCGAGACCACTAGTACGG
ACC_PTS_1_FW	GCTTCAGGCCCCTTTTCCTT
ACC_PTS_1_RV	AAGGTTTGTTGACCAGCAACAGTA
ACC_PTS_2_FW	TCCAGTTACTGTTGCTGGTCAACA
ACC_PTS_2_RV	GATATCGACAAAGGAAAAGGGGCCTGAAGCTTACAACTTAGACAAA CCTTTCAGCAGTTCTGCT
POS5_PTS_FW	GATGGTCTCACATGATGTCCACTTTGGACTCCCATTCC
POS5_PTS_RV	GCTTAGGTCTCAAAGCTTACAACTTAGAGTCGTTGTCAGTCTGTCTCT TAGTCAATC
